# Common Serum Biomarkers and Combination Ratios in the Diagnosis of Periprosthetic Joint Infection Following Total Hip Arthroplasty

**DOI:** 10.3390/microorganisms14020461

**Published:** 2026-02-13

**Authors:** Jason M. Dayan, Don H. Le, Anzar Sarfraz, Theodor Di Pauli von Treuheim, Farouk Khury, Sallie Yassin, Vinay K. Aggarwal, Ran Schwarzkopf, Alan J. Dayan

**Affiliations:** 1 Department of Orthopedics, Downstate Medical School, 395 Lenox Road, Brooklyn, NY 11203, USA; 2 Department of Orthopedics, McGovern Medical School at UTHealth Houston, Houston, TX 77030, USA; 3 Department of Orthopedics, New York University, New York, NY 10003, USA; 4 Division of Orthopedic Surgery, Rambam Health Care Campus, The Ruth and Bruce Rappaport Faculty of Medicine, Haifa 3109601, Israel

**Keywords:** periprosthetic joint infection (PJI), albumin, globulin, erythrocyte sedimentation rate (ESR), total protein, albumin–globulin ratio (AGR), CRP–albumin ratio (CAR), CRP-AGR ratio (CAGR)

## Abstract

Accurate preoperative diagnosis of periprosthetic joint infection (PJI) is difficult, complicating distinction between septic and aseptic failures. This study assessed the value of common serum biomarkers and three calculated ratios—albumin–globulin ratio (AGR), C-reactive protein–albumin ratio (CAR), and C-reactive protein–AGR ratio (CAGR)—in diagnosing PJI after primary total hip arthroplasty (THA). We retrospectively reviewed patients undergoing revision THA for PJI or aseptic failure from 2011 to 2021 at a single institution. Inclusion required reported serum white blood cell count (WBC), C-reactive protein (CRP), erythrocyte sedimentation rate (ESR), albumin (Alb), and total protein (TP). Diagnostic performance was evaluated using areas under the curve (AUCs), with higher values indicating better accuracy. Ratios were defined as: AGR = Alb/[TP − Alb], CAR = CRP/Alb, and CAGR = CRP/AGR. Among 128 cases, 67 were PJI and 61 aseptic. AUCs were: WBC (0.53), CRP (0.69), ESR (0.75), Alb (0.69), Glb (0.63), TP (0.53), AGR (0.72), CAR (0.70), and CAGR (0.71). Optimal cutoff, sensitivity, and specificity were: CRP (10.5, 0.76, 0.59), ESR (41.0, 0.70, 0.72), AGR (1.10, 0.64, 0.75), CAR (3.37, 0.73, 0.64), and CAGR (10.9, 0.75, 0.66). ESR, AGR, CAR, and CAGR demonstrated acceptable accuracy. These readily available markers and ratios may aid PJI diagnosis, supporting improved clinical decision-making.

## 1. Introduction

Periprosthetic joint infection (PJI) is a devastating complication of total joint arthroplasty (TJA) that has substantial health implications as well as a high economic burden. PJI may require multiple surgical interventions, as well as long-term antibiotic therapy and rehabilitation, and is projected to cost the USA $1.85 billion annually by 2030 [1,2,3]. Thus, the importance of diagnosing and treating PJI early cannot be overstated. Despite the severity of this complication, there continues to be no “gold standard” preoperative diagnostic test available. The current diagnostic guidelines for PJI include two major criteria and six minor criteria, such that the presence of either major criterion or a combination of some of the minor criteria would indicate PJI [4]. However, measuring these criteria is not always feasible or easily accessible. For example, although synovial fluid aspirations are commonly used for diagnosis of PJI, primary dry taps make up 46% of all joint aspirations and are less accurate in PJI diagnosis [5]. Further, the use of alpha-defensin, an inflammatory marker measured as part of a minor criterion for PJI diagnosis, is not available in every hospital or in patients with dry taps during synovial fluid collection [6]. Thus, there is a need to assess easily obtainable serum biomarkers that are widely available for use in screening for PJI.

C-reactive protein (CRP), albumin (Alb), and total protein (TP) are commonly measured and easily obtainable serum biomarkers that represent the body’s inflammatory and nutritional state and may be helpful in screening for PJI, particularly when treating patients with uncertain diagnosis of PJI in the setting of a dry aspirate. Importantly, CRP rises in response to infection and inflammation, while Alb and TP can be used to derive serum globulin (Glb) levels by the equation TP = Alb + Glb [7,8,9]. The relative concentrations of Alb and Glb are known to vary in response to IL-6 such that Alb production is suppressed while Glb production is stimulated [10]. Therefore, Alb and Glb can act as indirect measurements of IL-6, an inflammatory cytokine that has shown to be highly accurate in PJI diagnosis, yet difficult to measure directly. Further, ratios of these biomarkers, including albumin–globulin ratio (AGR), CRP–albumin ratio (CAR), and CRP–AGR ratio (CAGR), may be helpful in screening for PJI.

Prior studies describe the use of AGR to assess the probability of PJI in patients undergoing revision TKA and THA. However, much of the existing literature combines both revision TKA and THA in its analyses [8,11,12]. Thus, the goal of the present study is to evaluate the efficacy of these serum biomarkers and biomarker ratios in predicting the presence of PJI solely in patients undergoing revision THA. We aimed to assess a large range of serum values and ratios within a cohort exclusively undergoing revision THA, offering a more targeted evaluation of serum biomarkers in this population. This allows for greater relevance to THA-specific revision strategies and can potentially enhance applicability in clinical practice. We hypothesize that these biomarkers and ratios will show high accuracy in the diagnosis of PJI in patients undergoing THA.

## 2. Materials and Methods

### 2.1. Patients

**Figure 1:**
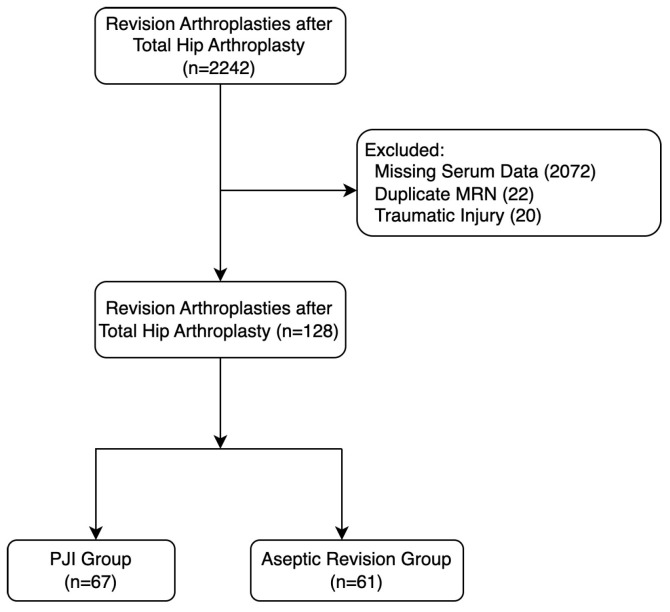
Patient enrollment flow diagram. “n” refers to the number of patients.

This study was approved by the institutional review board. We retrospectively analyzed a consecutive cohort of patients who underwent revision THA at our institution from 28 June 2011 to 11 January 2022. Inclusion criteria consisted of all patients who underwent index revision THA during this period with available preoperative serum biomarkers, including CRP, ESR, TP, Alb, and WBC levels within 2 months prior to the revision surgery. A total of 2242 patients were initially identified. Patients were excluded for the following reasons: missing preoperative biomarker data (2072 patients), duplicate medical record numbers (22 patients), and revision THA for traumatic injury (20 patients). Only patients with full preoperative data were included because calculation of the biomarker ratios required complete laboratory panels, making multiple imputation inappropriate. Therefore, a complete-case approach was used to handle missing data. Patients with systemic inflammatory diseases and those taking antibiotics were intentionally not excluded from this study, as IL-6 has demonstrated diagnostic reliability even in these settings and the serum biomarkers and ratios evaluated here function as indirect reflections of IL-6-mediated inflammatory activity [13,14,15,16,17]. This resulted in a final cohort of 128 patients who underwent index revision THA for either septic or aseptic indications (Figure 1 and Table 1). The diagnosis of PJI was determined by the treating surgeon in accordance with our institutional protocol, which follows the 2018 International Consensus Meeting criteria [4]. Patients were classified as having PJI or aseptic failure based on these criteria using available clinical, laboratory, microbiological, and intraoperative data. Culture-negative PJIs were included if they met the remaining ICM diagnostic criteria. There is no standardized protocol for obtaining preoperative serum biomarkers in cases where infection is not suspected, and the acquisition of these biomarkers was left to the discretion of the treating surgeon. Therefore, despite the high surgical volume, the lack of standardization in biomarker acquisition limited the availability of biomarker data, reducing the overall sample size. As a result, 67 patients were diagnosed with PJI (PJI group) and 61 patients were diagnosed with an aseptic revision (aseptic revision group). The mean ages of the PJI group and aseptic revision group were 62.5 (range: 29–85) and 66.1 (range: 24–90), respectively. Additionally, the percentages of women making up the PJI group and aseptic revision group were 34.3% and 54.0%, respectively (Table 1).

### 2.2. Measurements

All blood samples obtained within two months prior to surgery were directly measured per routine laboratory standards. CRP and ESR were measured as markers of inflammation, along with TP, Alb, Glb, and WBC as markers of immune response. Combination indices such as the Alb-Glb ratio (AGR), CRP-Alb ratio (CAR), and CRP-AGR ratio (CAGR) were also calculated to evaluate their utility in diagnosing PJI.

Preoperative serum inflammatory and immune marker levels were compared across the PJI and aseptic revisions. The data was analyzed to evaluate the performance of each serum biomarker for the diagnosis of infection, and comparisons were made to find the most accurate. Glb was calculated as Glb = TP − Alb and TP [7,8,9]. The diagnostic performance of these markers in identifying PJI was assessed using receiver operating characteristic (ROC) curves, with the area under the curve (AUC) serving as a measure of diagnostic accuracy. In general, an AUC of 0.5 suggests no discrimination (i.e., ability to diagnose patients with and without the disease or condition based on the test), 0.7 to 0.8 is considered acceptable, 0.8 to 0.9 is considered excellent, and more than 0.9 is considered outstanding [18]. The predictive capabilities of these markers were further analyzed by determining sensitivity, specificity, positive predictive value (PPV), and negative predictive value (NPV). An ROC curve analysis was performed for each marker to assess its diagnostic accuracy.

### 2.3. Data Analyses

Data analyses were conducted using R version 4.2.0 within the RStudio Posit Team 2025 environment [19]. Statistical significance was determined using Student’s *t*-tests, the Wilcoxon signed rank test, or one-way analyses of variance (ANOVA) for comparison between multiple groups. Although Student’s *t*-tests and ANOVA assume normally distributed data, they are robust to moderate deviations from normality, particularly with adequate sample sizes, similar group sizes and independent observations. Serum biomarkers such as CRP and ESR are known to demonstrate skewed distributions in clinical populations. Because of this expected non-normality, we selected statistical methods appropriate for nonparametric data when applicable, ensuring valid comparisons without formal normality testing. The diagnostic accuracy (AUC, sensitivity, specificity, positive predictive value, and negative predictive value) of the inflammatory, immune, and combination ratio biomarkers for the diagnosis of PJI was calculated for each marker based on a confirmed PJI diagnosis using R version 4.2.0 within the RStudio environment [19]. Cutoff values were determined using Youden’s index, calculated as Sensitivity + Specificity − 1, which identifies the cutoff point in the available data that maximizes the test’s ability to distinguish between positive and negative cases [20]. Manually selected cutoff values were used based on previously published cutoffs on the same serum biomarker ratios to compare changes in AUC, sensitivity, and specificity. Additional sensitivity tests were also performed while excluding extreme Alb and TP levels in the top and bottom 5% which resulted in unchanged AUCs. To determine whether differences in AUCs among biomarker ratios were statistically significant, we used a nonparametric bootstrap method with 2000 resamples. For each bootstrap sample, models were refit and evaluated in both the bootstrap and original datasets to estimate optimism. Optimism-corrected AUCs and calibration slopes were obtained by subtracting the average optimism from the apparent performance estimates. Differences in AUCs between models were assessed using bootstrap-derived confidence intervals. Given that laboratory assays have inherent variability, differences in AUCs may not necessarily reflect true clinical significance. This potential limitation was considered in our analysis and is discussed further in the limitations section.

## 3. Results

### 3.1. Demographics, Clinical Characteristics, and Serum Biomarker Values

**Figure 2:**
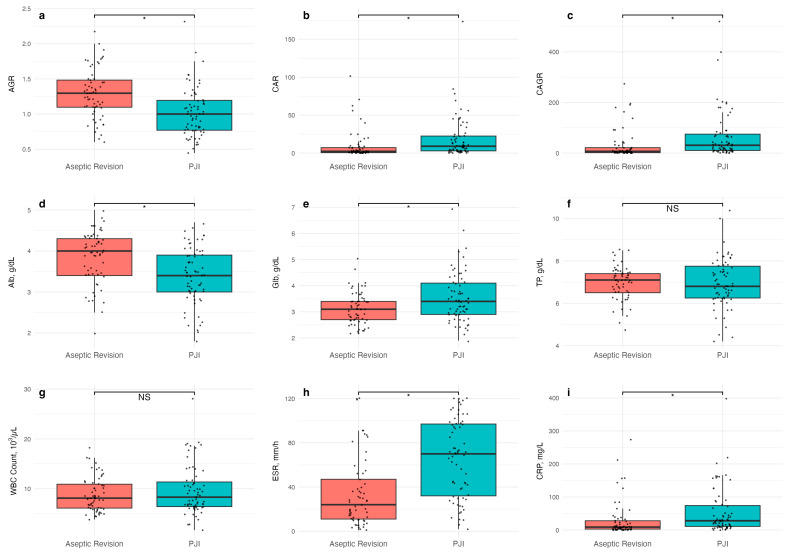
The difference in mean values of individual serum biomarkers and combination ratios. (**a**–**i**) Differences in values of serum markers of albumin–globulin ratio (AGR), CRP–albumin ratio (CAR), and CRP-AGR ratio (CAGR), albumin (Alb), globulin (Glb), total protein (TP), white blood cell count (WBC), erythrocyte sedimentation rate (ESR), and C-reactive protein (CRP) were compared among aseptic revision and periprosthetic joint infection (PJI) cases. * indicates a significant difference in mean values; NS, not significant.

The demographic data showed no significant differences in BMI, race, or smoking status between the two cohorts. However, the PJI group had a lower mean age and higher ratio of female patients compared to the aseptic revision group. Also, the PJI sample size was 67 patients while the aseptic revision sample size was 61 patients (Table 1). The single serum inflammatory markers CRP and ESR were both significantly higher in the PJI group than the aseptic revision group (*p* = 0.029 and *p* < 0.0001, respectively), while the WBC count was statistically unchanged (*p* = 0.279). Regarding single serum immune markers, Alb was significantly lower (*p* < 0.001) and Glb was significantly higher (*p* = 0.002) in the PJI group compared to the aseptic revision group, while total protein was statistically unchanged (*p* = 0.807). Regarding combination serum biomarker ratios, CAR and CAGR were significantly higher (*p* = 0.032 and *p* = 0.012, respectively), while AGR was significantly lower in the PJI group compared to the aseptic revision group (*p* <0.0001) (Figure 2 and Table 2).

### 3.2. Accuracy of Single Serum Biomarkers in the Diagnosis of Periprosthetic Joint Infection

**Figure 3:**
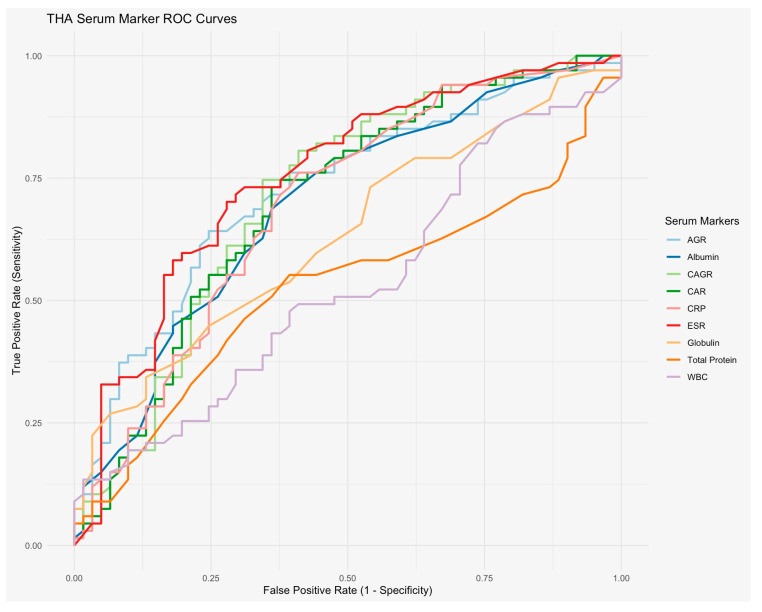
Diagnostic accuracy of individual serum biomarkers and combination ratios. The diagnostic values of CRP, ESR, AGR, CAR, and CAGR were all acceptable, with AUC values of 0.69 (CI 0.60–0.79), 0.75 (CI 0.67–0.84), 0.72 (CI 0.64–0.81), 0.70 (CI 0.61–0.79), and 0.71 (CI 0.62–0.80) for CRP, ESR, AGR, CAR, and CAGR, respectively.

Of the single serum inflammatory markers, ESR showed acceptable diagnostic accuracy with an AUC value of 0.75 (CI 0.67–0.84), while CRP and WBC count showed below acceptable accuracy with AUCs of 0.69 (CI 0.60–0.79) and 0.53 (0.43–0.63), respectively (Figure 3 and Table 3). Additionally, all three single serum immune markers showed below acceptable diagnostic accuracy, with AUC values of 0.69 (CI 0.60–0.79), 0.63 (CI 0.54–0.73), and 0.53 (CI 0.42–0.63), for Alb, Glb, and total protein, respectively (Figure 3 and Table 3).

### 3.3. Accuracy of Combination Serum Biomarker Ratios in the Diagnosis of Periprosthetic Joint Infection

All three combination serum biomarker ratios showed acceptable diagnostic accuracy, with AUC values of 0.72 (CI 0.64–0.81), 0.70 (CI 0.61–0.79), and 0.71 (CI 0.62–0.80) for AGR, CAR, and CAGR, respectively (Table 3 and Figure 2). For AGR, the ideal cutoff was 1.10 with a sensitivity of 0.64 (CI 0.39–0.76) and a specificity of 0.75 (CI 0.56–0.85). For CAR, the ideal cutoff was 3.37 with a sensitivity of 0.73 (CI 0.51–0.85) and a specificity of 0.64 (CI 0.44–0.75). For CAGR, the ideal cutoff was 10.9 with a sensitivity of 0.75 (CI 0.49–0.87) and a specificity of 0.66 (CI 0.46–0.77) (Table 3).

## 4. Discussion

The currently accepted definition of PJI, issued by the Musculoskeletal Infection Society (MSIS) and modified by the International Consensus Group, consists of two major criteria and six minor criteria [4,21,22]. Our study evaluated the diagnostic utility of individual serum biomarkers and combination ratios for identifying PJI. While CRP and ESR were significantly elevated in the PJI group, only ESR achieved acceptable diagnostic accuracy (AUC = 0.75). In contrast, other single markers including WBC, albumin, globulin, and total protein showed limited diagnostic value (AUC < 0.70). Notably, all three tested combination ratios (AGR, CAR, CAGR) demonstrated acceptable diagnostic accuracy, with AGR achieving the highest specificity (75%) at an optimal cutoff of 1.10. CRP and ESR elevation are currently included as minor criteria for PJI diagnosis. The role of CRP in the innate immune response, particularly in pathogen recognition and clearance, makes it valuable in the early detection of PJI. In uncomplicated THA and TKA, CRP typically peaks 2–3 days after surgery and normalizes within 3–6 weeks [16,23]. Thus, regular monitoring of CRP can assist in distinguishing between normal postoperative inflammation and complications such as PJI, facilitating timely interventions to prevent further complications. Apart from the known criteria, globulin was a particular focus of this study, representing a key marker of the systemic inflammatory response driven by IL-6 activity. IL-6 has been shown to be an excellent biomarker in PJI diagnosis even in patients taking antibiotics and patients with systemic inflammatory diseases [13,14,15,16,17]. However, IL-6 is not a routinely measured biomarker, likely owing to high costs and institutional limitations. IL-6 induces an extensive range of acute phase reactants, including CRP, serum amyloid A, fibrinogen, haptoglobin, and α1-antichymotrypsin, as well as immunoglobulin production in B cells, while reducing the production of albumin, fibronectin, and transferrin [10]. Consequently, studies have shifted toward indirect strategies to evaluate IL-6, leveraging its downstream effects on other routinely measured biomarkers [8,11,12,24].

Our study evaluated the diagnostic utility of individual serum biomarkers and combination ratios for identifying PJI. While CRP and ESR were significantly elevated in the PJI group, of the single serum biomarkers used in this study, only ESR showed acceptable diagnostic accuracy, with an AUC value in the 0.7 to 0.8 range, while the other measured single serum biomarkers fell below this range. Regarding the combination serum biomarker ratios used in this study, AGR, CAR, and CAGR all showed acceptable diagnostic accuracy, with AGR achieving the highest specificity (75%) at an optimal cutoff of 1.10, with AUC values in the 0.7 to 0.8 range. However, no single serum biomarker or combination ratio displayed AUC values in the excellent (0.8 to 0.9) or outstanding (greater than 0.9) range. These findings are interesting because a similar analysis from our institution on the utility of the studied biomarkers and combination ratios in PJI diagnosis was performed on a cohort of TKA patients and found better diagnostic accuracy [25].

Regarding single serum inflammatory markers, analysis of TKA performed at our institution found CRP and ESR to have AUC values of 0.85 and 0.76, respectively, compared to 0.69 and 0.72, respectively, in the THA analysis, although both analyses found WBC to display below acceptable diagnostic accuracy [25]. Regarding single serum immune markers, analysis of TKA patients found Alb to have an AUC value of 0.81, compared to 0.69 in the THA analysis, although both analyses found Glb and TP to display below acceptable diagnostic accuracy [25]. Comparison of these results shows that of the single serum biomarkers tested, CRP and Alb displayed superior diagnostic accuracy in TKA patients compared with THA patients, although CRP still had acceptable diagnostic accuracy in THA patients. Regarding the combination serum biomarker ratios, previous analysis of TKA patients found AGR, CAR, and CAGR to have AUC values of 0.78, 0.87, and 0.87, respectively, compared to AUC values of 0.72, 0.70, and 0.71, respectively, in the THA analysis [25]. Comparison of these results shows that while the studied combination ratios all show acceptable diagnostic accuracy in THA and TKA, CAR and CAGR displayed far superior diagnostic accuracy in TKA. It should also be noted that the ideal cutoffs for CAR and CAGR differed between the THA and TKA analyses such that the ideal cutoffs for CAR were 3.37 and 2.46 for THA and TKA, respectively, and the ideal cutoffs for CAGR were 10.9 and 7.09 for THA and TKA, respectively [25]. Nevertheless, we still recommend CRP, ESR, Alb, and the studied combination ratios to be evaluated as serologic tools for PJI screening and diagnosis, although there may be differences in the utility of these measurements between THA and TKA. This can be elucidated further through future prospective studies.

Other studies in the literature also reported encouraging results regarding the studied biomarkers and ratios in predicting PJI. One previous study by Dong et al. differentiated between THA and TKA patients and found that CRP, ESR, Glb, and AGR were all highly accurate in PJI diagnosis, with ESR, Glb, and AGR having AUC values above 0.8 and CRP having an AUC value above 0.9 for PJI diagnosis in THA, although their sample size was smaller and other ratios tested in this study were not analyzed [24]. Studies which did not separate THA and TKA groups and instead combined THA and TKA in their analyses reported high diagnostic accuracy for most biomarkers and all ratios, with AUC values falling in the excellent and outstanding ranges for ESR, CRP, Glb, CAR, and AGR and with AUC values falling in the acceptable and excellent ranges for Alb and CAGR [8,11,12]. These results differed from the results in this study, most notably for the single serum immune markers Alb and Glb, which displayed below acceptable diagnostic value in our analysis for patients following THA. A possible explanation for these differences is that our analysis included measurements taken up to 2 months prior to the revision surgery, compared to several of the cited studies who took measurements within a week of surgery [11,12,24], possibly negatively impacting our results. A longer period between serum measurements and the revision surgeries results in lowered sensitivity of the measured values in PJI diagnosis. Despite these variations in AUC values, the consensus in the available literature for common biomarkers and ratios in PJI diagnosis shows encouraging results. Therefore, we recommend that institutions regularly measure the studied biomarkers and ratios when there is suspicion for PJI, which will facilitate building larger sample sizes for future studies on this subject.

The variability in results between the THA and TKA analyses may simply be due to statistical limitations, such as smaller sample sizes or confounding variables, that may influence the results. Conversely, these differences may indicate more discrete properties of THA and TKA that must be further explored. One possible property that may influence the degree of immunologic and inflammatory response to infection, and therefore accuracy of biomarker measurements and combination ratios in PJI diagnosis, is differences in the profiles of infectious organisms most common in THA and TKA. The literature indicates that coagulase-negative Staphylococci account for a larger proportion of PJIs following THA than TKA, and that antibiotic-resistant organisms are also more frequently seen in THA [26,27,28]. Coagulase-negative Staphylococci are known to be less virulent and elicit a less aggressive immune response than other common infectious bacteria [29,30]. This can be explained by the fact that the main virulence factor of coagulase-negative Staphylococci is biofilm formation, which provides protection from external stresses including antimicrobial agents and host immune response, thus preventing excessive immune activation [29,31,32,33,34]. Further, the increased antimicrobial resistance exhibited in coagulase-negative Staphylococci can lead to a more chronic infection course, which is associated with decreased levels of acute immune and inflammatory markers, resulting in less accurate diagnostic value of these markers, as seen in the results of this study [30,35]. This contrasts with the pathogen profile more frequently observed in TKA-associated PJI, which may elicit a more robust inflammatory response and contribute to more pronounced variations in immune and inflammatory markers, leading to the stronger diagnostic performance of these biomarkers reported in our prior TKA cohort. Therefore, while the exact mechanism of immunologic variation between PJI in THA and TKA remains unclear, the possibility that unique characteristics exist between these two groups, THA and TKA, underscores the importance of using separate analyses when studying biomarker and combination ratios in PJI diagnosis for these two groups, THA and TKA patients, an approach that has been largely absent in prior studies for most of the studied biomarkers and ratios.

This study has several limitations. First, there was no standardized protocol for obtaining preoperative serum biomarkers in cases where infection was not suspected. As a result, the acquisition of these biomarkers was at the discretion of the attending surgeon. This variability may have introduced selection bias, as patients who appeared clinically concerning or had findings that raised any suspicion for PJI may have been more likely to undergo laboratory testing, potentially overrepresenting individuals at higher pre-test probability of infection. Moreover, the lack of a uniform protocol limits the reproducibility and broader applicability of our results, as differences in surgeon decision-making and patient assessment may not be consistent across institutions, and limited the overall sample size, potentially affecting the generalizability of our findings. Despite this, our cohort represents real-world conditions by mirroring the heterogeneity observed in routine practice, where decisions regarding laboratory tests are based on each surgeon’s clinical judgment and experience, and where differences in surgeon decision-making and patient characteristics naturally exist. Second, despite the high volume of revision THAs performed at our institution, individual serum biomarker testing was infrequent during the study period, which resulted in a smaller sample size than initially anticipated. Only 128 patients from the initial 2242 patients who received revision arthroplasties following THA in the same timeframe were included in this study, thereby restricting the statistical power of this study and potentially affecting the generalizability of our findings. A pre hoc power analysis was not conducted to determine the optimal sample size, which may have limited the ability to detect small differences between biomarkers. Nevertheless, previous studies, which often combined TKA and THA, also faced comparable or smaller sample groups [8,12,24]. Third, while diagnostic classification followed established institutional criteria, the absence of formal blinding may introduce bias and represents an inherent limitation of retrospective analyses. However, diagnostic classification was based on objective clinical, microbiological, and intraoperative criteria rather than the serum biomarkers under investigation, which mitigates the likelihood that lack of blinding materially influenced case assignment. Fourth, serum biomarkers—particularly albumin-based ratios (AGR, CAR, CAGR)—may be influenced by non-infectious factors such as nutritional status, chronic organ disease, malignancy, and baseline inflammatory or metabolic conditions, which were not adjusted for in multivariable analyses. These factors were intentionally incorporated to preserve real-world generalizability but may introduce residual confounding. Finally, the study is also limited by the inherent variability in laboratory measurement techniques for serum biomarkers. Differences in assay methods, reagent quality, and inter-laboratory calibration may introduce measurement errors that could influence the calculated values of the serum biomarkers and ratios. Nevertheless, our findings suggest that the assessed serum markers have diagnostic value and provide a foundation for future prospective studies with larger sample sizes and higher statistical power to further validate and add to our findings. Future studies should consider multicenter validation to assess the reproducibility of the ratios used in this study across different laboratory settings. Additionally, further investigation is needed to validate serum PJI diagnosis in patients with complex comorbidities or who are concurrently taking various antibiotics. Therefore, a large-scale, multicenter, prospective study should be designed to evaluate the diagnostic accuracy of individual serum biomarkers, such as Alb, CRP, and ESR, as well as combined indices like CAR and CAGR, for the diagnosis of PJI in THA. Finally, because this study focused solely on THA, and past studies have focused on THA and/or TKA, additional studies should be done to verify serum biomarker utility in total shoulder arthroplasty to compare possible differences between those findings and findings for THA and TKA.

## 5. Conclusions

Combination serum biomarker ratios (AGR, CAR, CAGR) demonstrated acceptable diagnostic accuracy for distinguishing septic from aseptic revision. AGR, in particular, showed the strongest discriminatory performance among these ratios. While single biomarkers like ESR also exhibited somewhat adequate accuracy, combining these readily available biomarkers into ratios can help in terms of early and improved diagnostic value and can be used as supplementary tools. Prospective studies with larger sample sizes should be performed to further validate our findings.

## Figures and Tables

**Table 1:** Patient demographic data.

Group	PJI (n = 67)	Aseptic Revisions (n = 61)	*p*-Value (Wilcoxon)
BMI (Range)	30.8 (18–57)	28.7 (18–52)	0.052
Age (Range)	62.5 (29–85)	66.1 (24–90)	0.009
Female (%)	34.3%	54.0%	0.038
Race (%)			0.559
White	73%	74%	
African American (Black)	13%	7%	
Other Race	13%	17%	
Patient Refused	N/A	2%	
Smoking Status (%)			0.438
Never Smoker	42%	51%	
Former Smoker	45%	36%	
Current Smoker	13%	13%	

PJI, periprosthetic joint infection; BMI, body mass index.

**Table 2:** Mean (standard deviation in parentheses) values of serum markers.

Serum Laboratory Data	PJI	Aseptic Revisions	*p*-Value (Student’s *t*-Test)
Inflammatory Markers			
WBC Count, 10^3^/µL	9.64 (4.91)	8.83 (3.41)	0.279
CRP, mg/L	55.6 (70.5)	31.02 (54.87)	<0.05
ESR, mm/h	65.3 (35.3)	34.51 (31.73)	<0.0001
Immune Markers			
Alb, g/dL	3.4 (0.69)	3.87 (0.63)	<0.05
Glb, g/dL	3.55 (0.95)	3.12 (0.61)	<0.05
TP, g/dL	6.95 (1.17)	6.99 (0.79)	0.807
Combination Ratios			
AGR	1.02 (0.35)	1.29 (0.35)	<0.0001
CAR	19.1 (27.7)	9.96 (19.5)	<0.05
CAGR	68.6 (99.3)	31.9 (58.7)	<0.05

PJI, periprosthetic joint infection; WBC, white blood cell; CRP, C-reactive protein; ESR, erythrocyte sedimentation rate; Alb, albumin; Glb, globulin; TP, total protein; AGR, albumin/globulin ratio; CAR, CRP–albumin ratio; CAGR, C-reactive protein–AGR ratio.

**Table 3:** Diagnostic accuracy of individual serum markers and combination ratios for PJI.

Diagnostic Accuracy	AUC	Cutoff	Sensitivity	Specificity	PPV	NPV
Inflammatory Markers						
WBC Count (10^3^/µL)	0.53 (0.43–0.63)	16.3 ^a^	0.13 (0.04–0.22)	0.98 (0.82–1.00)	0.90 (0.60–0.98)	0.51 (0.56–0.60)
CRP (mg/L)	0.69 (0.60–0.79)	10.5 ^a^	0.76 (0.52–0.87)	0.59 (0.38–0.72)	0.67 (0.56–0.77)	0.69 (0.56–0.80)
		6 ^b^	0.90 (0.80–0.95)	0.34 (0.24–0.47)	0.60 (0.50–0.69)	0.75 (0.50–0.69)
		10 ^c^	0.76 (0.65–0.85)	0.56 (0.43–0.67)	0.65 (0.54–0.75)	0.68 (0.54–0.79)
ESR (mm/h)	0.75 (0.67–0.84)	41.0 ^a^	0.70 (0.51–0.82)	0.72 (0.52–0.84)	0.72 (0.62–0.83)	0.69 (0.57–0.79)
		30 ^c^	0.79 (0.68–0.87)	0.57 (0.45–0.69)	0.67 (0.56–0.76)	0.71 (0.58–0.82)
Immune Markers						
Alb (g/dL)	0.69 (0.60–0.79)	3.80 ^a^	0.69 (0.47–0.83)	0.64 (0.46–0.76)	0.35 (0.24–0.48)	0.32 (0.22–0.44)
Glb (g/dL)	0.63 (0.54–0.73)	3.75 ^a^	0.34 (0.19–0.48)	0.87 (0.69–0.97)	0.74 (0.57–0.86)	0.55 (0.45–0.64)
TP(g/dL)	0.53 (0.42–0.63)	6.95 ^a^	0.55 (0.37–0.66)	0.61 (0.23–0.75)	0.45 (0.33–0.57)	0.39 (0.28–0.52)
Combination Ratios						
		1	0.35 (0.25–0.48)	0.26 (0.17–0.38)	0.35 (0.25–0.47)	0.27 (0.17–0.40)
AGR	0.72 (0.64–0.81)	1.1 ^a^	0.64 (0.39–0.76)	0.75 (0.56–0.85)	0.34 (0.24–0.46)	0.26 (0.16–0.38)
		1.2 ^b^	0.25 (0.16–0.37)	0.39 (0.28–0.52)	0.31 (0.21–0.45)	0.32 (0.23–0.44)
CAR	0.70 (0.61–0.79)	3.37 ^a^	0.73 (0.51–0.85)	0.64 (0.44–0.75)	0.69 (0.58–0.79)	0.68 (0.56–0.79)
CAGR	0.71 (0.62–0.80)	10.9 ^a^	0.75 (0.49–0.87)	0.66 (0.46–0.77)	0.70 (0.59–0.80)	0.70 (0.57–0.80)

Numbers in parentheses indicate 95% confidence intervals. AUC, area under the curve; PPV, positive predictive value; NPV, negative predictive value; WBC, white blood cell; CRP, C-reactive protein; ESR, erythrocyte sedimentation rate; Alb, albumin; Glb, globulin; TP, total protein; AGR, albumin/globulin ratio; CAR, CRP–albumin ratio; CAGR, C-reactive protein–AGR ratio. ^a^ Youden’s index [20]. ^b^ Previously published cutoffs [8]. ^c^ ICM serum criteria for chronic PJI [4].

## Data Availability

The original contributions presented in this study are included in the article. Further inquiries can be directed to the corresponding author.
